# Random Forest and SHAP Analysis on Social Vulnerability and Edentulism among US Adults 65 and Older

**DOI:** 10.1093/geroni/igaf122.4181

**Published:** 2025-12-31

**Authors:** Jinbo Niu, Joonhyeog Park, Tamara Cadet

**Affiliations:** University of Southern California, Los Angeles, California, United States; University of Pennsylvania, Philadelphia, Pennsylvania, United States; University of Pennsylvania, Philadelphia, Pennsylvania, United States

## Abstract

Approximately 17.3% of adults aged 65 and older in the U.S. face complete edentulism. Identifying the social factors that contribute to edentulism and their interactions is crucial for creating predictive models and effective interventions. However, most studies ignored the interactions among social factors on the edentulism prevalence. Using the concept of social vulnerability, we integrated the latest CDC Places data (2022) and Social Vulnerability Index (SVI) data (2022) to analyze the prevalence of edentulism among adults aged 65 and older across over 3,000 U.S. counties. Random Forest (RF) and SHapley Additive exPlanations (SHAP) were applied to identify the main driving factors of edentulism and characterize their interaction effects. Among the factors considered, the poverty rate emerged as the most influential in predicting edentulism prevalence, followed by the no-high-school-diploma rate and the internet inaccessibility rate. The interaction between poverty rate and no-high-school-diploma rate had the strongest effect on edentulism prevalence. On average, the interactions between social vulnerability factors accounted for 123.08% of the direct influence on edentulism prevalence. The analysis also revealed critical thresholds where the combined impact of these factors shifts to either exacerbate or moderate edentulism prevalence. Social vulnerabilities do not operate in isolation, and their interactions can exacerbate or moderate edentulism among older adults. Accounting for interaction effects and thresholds can refine prediction modeling and guide integrated interventions addressing poverty, education, and internet access to better target high-risk communities.

